# Chytridiomycosis infection and heat compromise sperm quality in a threatened frog

**DOI:** 10.1242/jeb.252760

**Published:** 2026-08-04

**Authors:** Rose Upton, Anne Ibbotson, Kaya Klop-Toker, Lachlan Campbell, Nadine Nolan, Phillip Jobling, Michael Mahony, John Clulow, Natalie E. Calatayud, Alex Callen

**Affiliations:** ^1^ Amphibian Integrated Conservation Unit, Centre for Conservation Science, School of Environmental and Life Sciences, The University of Newcastle, Callaghan, NSW 2308, Australia; ^2^ School of Biomedical Sciences and Pharmacy, The University of Newcastle, Callaghan, NSW 2308, Australia; ^3^ Museums Victoria Research Institute, Carlton, Victoria 3053, Australia

**Keywords:** Phenotypic plasticity, Terminal investment, Reproductive–immune trade-off, Thermal stress, Spermatogenesis

## Abstract

Environmental change is reshaping wildlife reproduction through increasing temperatures and often leads to the spread of emerging infectious diseases, yet the physiological consequences of managing these stressors remain poorly understood. Amphibians are particularly vulnerable owing to their ectothermy and high susceptibility to chytridiomycosis, caused by *Batrachochytrium dendrobatidis* (*Bd*). Here, we examined how *Bd* infection and thermal treatment interact to influence sperm quality and reproductive investment in male green and golden bell frogs (*Ranoidea aurea*), a species that has suffered severe population declines. Moderate *Bd* infection was associated with elevated sperm concentration relative to uninfected and heavily infected males, consistent with increased short-term reproductive investment under elevated mortality risk. However, severe infection led to pronounced reductions in sperm concentration and motility. Thermal treatment successfully eliminated *Bd* infection but imposed reproductive costs: sperm concentration declined following treatment and remained significantly reduced 6 months later, despite partial recovery of sperm motility and membrane integrity. Our findings reveal that disease and thermal stress jointly shape amphibian reproductive outcomes through context-dependent trade-offs between immune defence and gamete production. Although mild infection may trigger short-lived increases in reproductive output, both severe infection and pathogen clearance via thermal exposure impose lasting constraints on fertility. These results highlight an underappreciated cost of disease mitigation and suggest that increasing thermal extremes associated with climate change may further limit amphibian reproductive resilience, with important implications for conservation management and population persistence.

## Teaser

**Highlighted Article:** Moderate chytrid infection may temporarily boost reproductive investment in a threatened frog, but severe infection and heat-based disease treatment cause lasting declines in sperm quality and fertility.

## INTRODUCTION

Environmental change is reshaping wildlife health and reproduction through shifts in temperature and altered precipitation, leading to the spread of emerging diseases. These stressors disrupt endocrine and immune systems, leading to reduced fertility, abnormal development and increased disease susceptibility across vertebrates (Acevedo-Whitehouse and Duffus, 2009). Global assessments show that amphibians are among the most affected, with disease and climate change together driving the majority of recent declines (Luedtke et al., 2023). As ectotherms, amphibians depend heavily on environmental temperature and moisture to regulate physiological function, making them particularly vulnerable to rapid climate variability. Recent analyses indicate that global warming is already pushing several species beyond their physiological heat limits, even within shaded or aquatic habitats that were once thermally stable (Pottier et al., 2025). These findings highlight how increasing temperature extremes and disease outbreaks act together to constrain amphibian survival in a rapidly changing world (Calatayud et al., 2024).

The ability of vertebrates to cope with such instability depends on phenotypic plasticity, the capacity to adjust physiological and behavioural traits to maintain homeostasis and reproductive function under changing conditions (Canale and Henry, 2010). In amphibians, this plasticity is central to balancing immune defence, breeding effort and energetic costs across fluctuating environments. Yet, the limits of plasticity become apparent when environmental change outpaces physiological compensation, as occurs under combined heat and disease stress. In such cases, reproductive and immune processes can become misaligned, resulting in reduced gamete production, impaired fertility and compromised resilience (Park and Do, 2023; Schmidt et al., 2024). As outlined in the Amphibian Conservation Action Plan, these physiological thresholds and the limited capacity of many amphibians to adapt or shift their ranges underscore the urgency of understanding reproductive resilience under compounding stressors (Calatayud et al., 2024).

Among the most devastating examples of how disease and climate interact to threaten biodiversity is the emergence and persistence of chytridiomycosis, caused by the fungal pathogen *Batrachochytrium dendrobatidis* (*Bd*). This pathogen has driven the extinction of more than 90 amphibian species globally, including at least seven in Australia, and continues to contribute to the decline of more than 500 others, making it one of the most destructive infectious agents known to science (Berger et al., 1998; Scheele et al., 2019; Skerratt et al., 2016). The relationship between *Bd* and temperature is complex: infection thrives in cool, moist environments, whereas higher temperatures can suppress the pathogen but impose physiological strain on the host (Hamer et al., 2003; Waddle et al., 2024). With climate warming predicted to increase the frequency of extreme thermal events, amphibians now face overlapping pressures of thermal stress and persistent infection, each influencing immune competence and reproductive performance (Park and Do, 2023).

The green and golden bell frog, *Ranoidea aurea* (previously *Litoria aurea*), exemplifies this intersection between disease, physiology and climate vulnerability. Once widespread along the southeastern coast of Australia, the species has suffered extirpations across more than 90% of its range over recent decades (Mahony et al., 2013). Chytridiomycosis is considered the primary driver of its decline (Abu Bakar et al., 2016; Stockwell et al., 2008), and few management options exist for wild populations (but see Clulow and Clulow, 2016). Because *R. aurea* can be maintained successfully in captivity and responds well to assisted reproductive technologies (Clulow et al., 2018; Upton et al., 2024, 2021), it provides an ideal system for investigating the physiological effects of disease and potential management strategies recently trialled, including vaccination and heat-based infection clearance (Waddle et al., 2024). These management approaches reflect the integrated conservation frameworks promoted by the Amphibian Conservation Action Plan (Calatayud et al., 2024), which emphasize coordination between *ex situ* population management, disease control and reproductive technologies.

Conservation strategies have predominantly focused on preventing extinction through the establishment of quarantined, disease-free colonies and through controlled thermal treatments aimed at clearing infection, but there is little research examining the interactions between reproductive plasticity, disease, and thermal tolerance and recovery. Linking these management strategies to their physiological consequences provides essential insight into how amphibians balance infection control with the maintenance of reproductive capacity. Elevated temperatures are known to disrupt spermatogenesis through oxidative stress, mitochondrial dysfunction and germ-cell apoptosis in many vertebrates (Agarwal et al., 2014; Houston et al., 2018), and comparable effects are expected in ectotherms. Such cellular stress pathways may underlie the trade-offs observed in amphibians undergoing thermal treatment, where infection clearance can coincide with declines in reproductive capacity (Campbell et al., 2019).

The energetic conflict between immune defence and reproduction provides a theoretical foundation for understanding these trade-offs. The terminal investment hypothesis predicts that individuals facing reduced survival prospects will allocate resources toward immediate reproductive effort rather than future survival (Clutton-Brock, 1984; Duffield et al., 2017; Williams, 1966). Although this response has been documented across diverse taxa (Duffield et al., 2018; Foo et al., 2023), amphibians offer a unique system in which to test how infection and environmental stress interact to shape reproductive investment. Evidence from *Bd*-infected species shows variable outcomes: some populations exhibit increased reproductive effort (Brannelly et al., 2016, 2021), whereas others show reduced gonadal investment and sperm quality (Chatfield et al., 2013; Kelleher et al., 2021). This variation reflects the context-dependent and plastic nature of reproductive responses to infection severity and thermal environment.

This study examines how *Bd* infection and elevated temperature interact to influence sperm quality and reproductive investment in male *R. aurea*, a species that has undergone severe declines due to chytridiomycosis. We specifically aimed to: (i) determine how variation in *Bd* infection severity affects sperm quality; (ii) assess the effects of initial infection status and heat-based pathogen clearance on sperm quality; and (iii) quantify the short- and long-term consequences of thermal treatment for sperm quality, including effects persisting 6 months post-treatment. By explicitly linking infection burden, disease mitigation and recovery time, this study provides mechanistic insight into how immune activation and thermal stress regulate reproductive trade-offs and constrain amphibian resilience under ongoing climate change.

## MATERIALS AND METHODS

### Study species and animal husbandry

All animals in this study were sourced from the University of Newcastle's *Ranoidea aurea* (Lesson 1829) captive breeding colony. Frogs were housed in seminatural outdoor mesocosms (75×75×110 cm) containing approximately 25% area terrestrial gravel substrate with vegetation, 75% water and stacked brick microrefuges throughout. Frogs were fed with crickets one to two times per week, supplemented with calcium and vitamin powder (Multical Dust; Vetafarm, Wagga Wagga, NSW, Australia), and exposed to natural day–night light regimes, being able to bask at will. Frogs were transferred to the laboratory for heat treatment from the outdoor holding mesocosms in July 2024 after *Bd* was detected during disease surveillance. Once indoors, frogs were group housed in plastic terraria (30×18×20 cm), with 25% terrestrial environment (autoclaved pebbles) and 75% aquatic environment (aged tap water) and plastic piping as hides, with four frogs per plastic terrarium.

All frogs underwent a precautionary protocol of heat treatment regardless of quantitative polymerase chain reaction (qPCR) results to ensure no *Bd* infection remained in the colony and opportunistic sampling was performed, as outlined below. Frogs were placed in UV- and temperature-controlled cabinets (TRISL – 1175, Thermoline Scientific Equipment, Wetherill Park, NSW, Australia) on a 12 h:12 h UV light cycle started at 25°C. Temperature was increased by 2°C per day until 37°C was reached, where the temperature was held for 6 h. This temperature has been shown to kill *Bd* within 4 h (Johnson et al., 2003; Upton et al., 2024; Woodhams et al., 2003). Subsequently, temperature was reduced by 2°C per day until the original 25°C was reached and frogs were reswabbed to confirm disease had been cleared. Frogs were kept at 24–25°C until the conclusion of the experiment.

All captive husbandry and experiments were conducted in accordance with institutional and national Australian standards for the care and welfare of animals, and in accordance with the University of Newcastle's animal ethics approval (A-2022-207 and A-2024-413). Animals were originally collected under NSW Scientific Licence SL101269.

### Opportunistic sampling

Although infection of the frogs in this study was not part of a planned experiment, the outbreak event allowed opportunistic sampling to investigate the effects of disease and treatment on sperm quality. On the same day, but prior to commencing heat treatment, all frogs were weighed (to nearest 0.1 g) and snout–vent length (SVL) and right tibia were measured (to the nearest 0.1 mm). All frogs were swabbed to determine *Bd* infection rates and loads via qPCR. Swabs were stored at −30°C until DNA extraction. Forty-two frogs in total were affected by this outbreak, with six deaths (two male, four female) due to *Bd*. Of the remaining 36 frogs, 25 were male and 11 were female. As these adults were sourced from wild populations, ages were unknown. On day 2 of heat treatment, a subset of 13 mature males was selected for sperm induction, blood collection and biopsy of the toe webbing. All frogs that were selected were without advanced symptoms and had consistent and prompt righting reflexes of under 1 s. In addition, two males that were found deceased at the discovery of the outbreak were dissected, and sperm quality from testicular macerates was assessed (see ‘Induction of spermiation and sperm collection’ below).

#### Induction of spermiation and sperm collection

Frogs were removed from the heat treatment in the morning, prior to increasing the temperature for the day, for 2 h and kept at 25°C. After urine samples containing sperm (spermic urine) were collected, animals were returned to the heat treatment and the temperature was raised to 27°C. As frogs were originally sourced from wild populations, age was unknown; however, males reach sexual maturity at around 1 year of age and all displayed secondary sexual characteristics indicative of sexual maturity (darkened nuptial pads and coloured throat). The mass range of males used in the study was 21.5±3.8 g (mean±s.e.m., *n*=13). None of the 13 males selected for spermiation experiments died due to infection.

A sample of urine was collected via catheterization (described below) prior to hormone injection and found to be aspermic. Males were injected with 5 IU g^−1^ body mass human chorionic gonadotropin. Previous studies have shown no difference in sperm concentrations yielded between doses of 5, 10 and 20 IU g^−1^ body mass, and recent data have refined the protocol to use less hormone per injection (R.U., unpublished data). Hormones were diluted in simplified amphibian ringer (SAR; 113 mmol l^−1^ NaCl, 1 mmol l^−1^ CaCl2, 2 mmol l^−1^ KCl, 3.6 mmol l^−1^ NaHCO3; ∼220 mOsm kg^−1^; recipe from Browne et al., 1998) to a final volume of 200 μl. Hormones were administered subcutaneously via the dorsal lymph sac using a 31-gauge insulin syringe (BD, NJ, USA). Frogs were placed in individual inflated plastic bags with aged tap water sprayed into the bag for hydration. Half an hour after injection, a urinary catheter (Tomcat feline catheter; Argyle) was used to collect spermic urine samples (and pre-injection sperm samples). The tip was placed into the cloaca and gently moved in and out to facilitate urine collection by capillary action. Spermic urine was collected at intervals of 10–15 min over an hour period and pooled in a 1.5 ml Eppendorf tube for analysis. Samples were kept on ice until volume, motility and sperm concentration could be determined (within the same day). Motility analyses were performed immediately after collection to reduce possible effects of time post-collection, followed by assessment of membrane integrity and sperm concentration.

In two frogs found recently deceased (not included in sperm induction experiment), both testes were removed and placed in 1 ml SAR. A sperm suspension was prepared by gently teasing apart testicular tissue with fine forceps to release sperm. Sperm quality assessments, described below, were conducted immediately after maceration.

#### Post-treatment sampling

Sperm sampling was repeated 5 weeks following the conclusion of heat treatment (7 weeks following initial outbreak detection), in September 2024. Although the duration of spermatogenesis is unknown in this species, previous work has shown the duration of spermatogenesis to be approximately 40 days in *Lithobates catesbeianus* (Segatelli et al., 2009). Thus, to determine whether any short-term effects of *Bd* infection were still present, this period was chosen to allow a full cycle of spermatogenesis to occur post-infection. As results indicated a possible effect of heat treatment on sperm quality, a subset of four of the 13 frogs were sampled again 6 months following completion of heat treatment. The full cohort was unable to be sampled at 6 months post-heat treatment owing to frogs entering breeding programmes. All four frogs sampled at 6 months were positive for *Bd* at their initial timepoint but were cleared of *Bd* by the initial heat treatment.

### DNA extraction and qPCR

A standardised swabbing technique was employed over the epidermal surfaces prone to high infection loads, which involved wiping both sides of the ventral skin 8 times, the inner and outer thighs 4 times, and the hind and fore feet 2 times each (Stockwell et al., 2010). DNA was extracted from swab tips and quantified following standard qPCR Taqman assay methods (Boyle et al., 2004) using a Bio-Rad CFX96 system. Each swab was assessed for the presence of *Bd* in triplicate using a 1/10 diluted sample from the DNA extracts. Standard curves were generated using commercially prepared standards (Pisces Molecular, Boulder, CO, USA) and were serially diluted to prepare five known *Bd* concentrations [18,700, 1870, 187, 18.7 and 1.87 genomic equivalents (GE) μl^−1^]. Final data were expressed as mean genomic equivalents per microlitre of a standardized extract volume (5 μl) from three replicates of the same swab sample. A frog was considered uninfected if the mean swab result was <1.00 zoospore equivalents (Klop-Toker et al., 2016), if at least two of the triplicates did not amplify (Campbell et al., 2019; Stockwell et al., 2010) or where amplification occurred >40 cycles, which was interpreted as non-specific binding. Where at least two triplicates amplified, the average of all three wells was used if no presence of reaction inhibition was present. Inhibition was detected with inclusion of an internal positive control (Taqman Exogenous Internal Positive Control Reagents; Applied Biosystems, MA, USA) in a single replicate of each sample. Where the internal control did not amplify, samples were considered inhibited and rerun at a 1:100 dilution. Data were multiplied by either 10 or 100 to account for the dilution step during the extraction process.

### Sperm quality assessments

Concentration (cells ml^−1^) of sperm in urine samples was assessed with an Improved Neubauer haemocytometer counting chamber at each collection interval. Sperm were diluted, as necessary, with 1.7% NaCl and the dilution factor was accounted for in the final calculation. Ten microlitres of sperm solution was pipetted into each of two chambers, and the number of sperm in at least five secondary quadrats (per chamber) was counted, such that at least 80–100 sperm cells were counted per sample, and used to calculate total sperm per millilitre. The number of secondary quadrats was accounted for in the final calculation.

Owing to high concentrations of spermic urine present in samples, motility was analysed in all samples after a 1:5 dilution in reverse osmosis (RO) water. Although analysis of undiluted samples was preferable, high densities of sperm made visualization of individual sperm cells impossible. Sperm were categorized as either non-motile, motile without forward progression or motile with forward progression, and at least 100 sperm were counted at 400× magnification under phase contrast optics. Proportions were calculated for forward-progressive and total motility (sperm motile with or without forward progression). Membrane integrity was determined using a dye-exclusion assay. Eosin-Y (Sigma Aldrich; E4009; 0.4% w/v diluted in 0.9% w/v saline) was mixed 1:1 with sperm samples, incubated for at least 30 s at room temperature and then scored under a coverslip using brightfield microscope at a magnification of 400×. Sperm that stained pink were scored as compromised, whereas sperm with a clear cytoplasm were scored membrane intact. For each sample, 100–200 sperm were scored per count, and two counts were conducted per sample. If two counts from the same sample differed substantially, a third count was made and included in the analysis.

### Statistics and experimental design

Generalized linear mixed models (GLMMs) were used to fit Poisson and binary logistic regressions to four sperm parameters: sperm concentration (cells ml^−1^), proportion forward-progressive motility, proportion total motile and proportion membrane intact. For sperm concentration, raw counts were used with an offset of the log reciprocal of an adjustment factor (based on the formula for calculating sperm concentration from a haemocytometer, accounting for: dilution factor, volume and the number of quadrats counted) to convert the raw counts to cells ml^−1^. For the motility and membrane integrity measures, the models were interpreted as the proportion of successful cases, i.e. live spermatozoa or motile sperm with the total number of spermatozoa used as weights in the model. For all models, outputs were calculated as estimated marginal means (EMMs) and 95% confidence intervals. EMMs were chosen because they represent model adjusted expected measures, while accounting for repeated measures (animal ID) and technical replicates (two per animal).

For experiment 1 (effect of *Bd* severity on sperm quality), *Bd* infection severity was used as a main effect, where the categories were: negative, 0 GE μl^−1^ (*n*=4); moderate, 900–6499 GE μl^−1^ (*n*=5); and high, >6500 GE  μl^−1^ (*n*=4). For experiment 2 (effect of initial *Bd* status on sperm quality pre- and post-treatment), a two-factor design was implemented, with *Bd* status prior to heat treatment (negative, *n*=4; positive, *n*=9) and time in relation to heat treatment (pre- and post-heat treatment) used as main effects. The interaction between initial *Bd* status and time in relation to heat treatment was not significant and was dropped from the model. For experiment 3 (effect of heat treatment on sperm quality), the time in relation to heat treatment was used as a main effect (pre-, post- and 6 months post-heat treatment) using a subset of four frogs from each time point. For all models, overdispersion was addressed using animal body condition (mass/SVL) as a random effect. Significance was determined by pairwise comparisons to determine ratios of expected counts between conditions and 95% confidence intervals. Modelled EMMs and 95% confidence intervals (CIs) were calculated.

All analyses were completed using R (version 4.4.0; https://www.r-project.org/) with the R package glmmTMB used for all GLMM modelling (Brooks et al., 2017). The package emmeans (https://CRAN.R-project.org/package=emmeans) was used to model EMMs, and 95% confidence limits were calculated and back-transformed to sperm concentrations and motility and membrane intact proportions. Ratios (for sperm concentration data) and odds ratios (for proportion data) comparing treatments were also generated using the package emmeans. All graphing was completed using ggplot2 (Wickham, 2016) and gridExtra (https://CRAN.R-project.org/package=gridExtra).

## RESULTS

### *Bd* load analysis

Four out of 13 males in this study tested negative for *Bd*. The nine remaining frogs had moderate to high loads of *Bd*, ranging from 939 to 28,010 GE μl^−1^ and averaging 7815 GE μl^−1^. Five frogs were categorized as moderately infected, with an average load of 1439 GE μl^−1^, and four frogs were categorized as highly infected with an average of 15,784 GE μl^−1^. Two deceased frogs from this study had 403,370 and 34,000 GE μl^−1^, respectively. Following heat treatment, all frogs tested negative for *Bd*.

### Experiment 1: effect of *Bd* severity on sperm quality

**Fig. 1.**
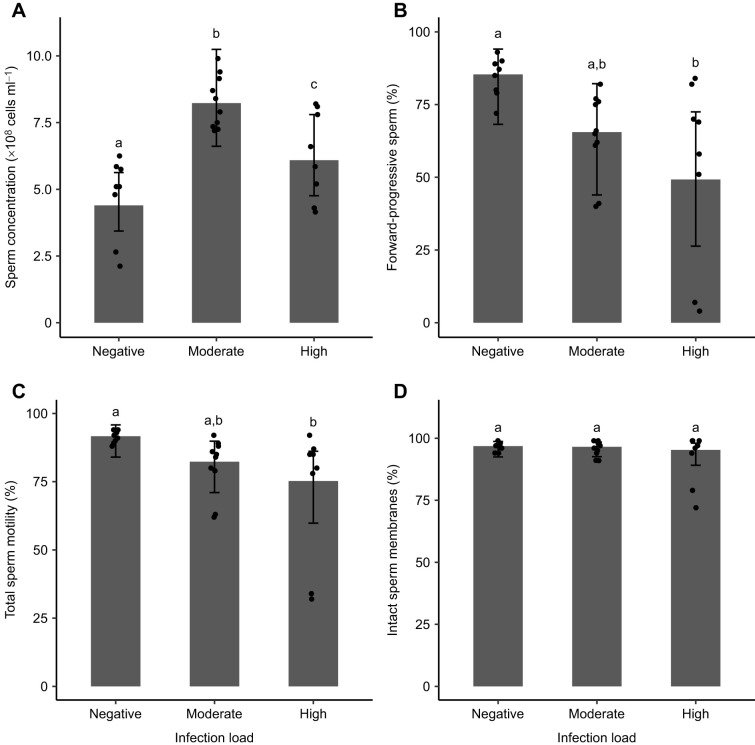
**Effect of *Bd* infection load on induction of spermiation in *Ranoidea aurea*.** (A) Sperm concentration (cells ml^−1^); (B) forward-progressive sperm (%); (C) total sperm motile (%); and (D) intact membranes (%). Infection severity categories: negative, 0 GE μl^−1^ (*n*=4); moderate, 900–6499 GE μl^−1^ (*n*=5); and high, >6500 GE μl^−1^ (*n*=4). Columns represent estimated marginal means, error bars equal 95% confidence intervals, and black dots represent raw data. Two technical replicates were made per frog, which was included as a random effect in the generalized linear mixed model. Different letters indicate significant differences among groups (*P*<0.05, *n*=13); groups sharing a letter are not significantly different.

There was a significant effect of *Bd* severity on sperm concentration [likelihood ratio test (LRT) χ^2^_2_=9.4, *P*=0.009], with frogs in the moderate infection category having higher sperm concentrations (EMM: 8.2×10^8^; 95% CIs: 6.6×10^8^, 1.0×10^9^) than high load infections and frogs negative for infection (Fig. 1A). Ratios and 95% CIs were generated to further compare the differences in sperm concentration in response to *Bd* infection, with larger ratios indicating bigger differences in sperm concentration. EMMs ranged from 4.4×10^8^ to 8.2×10^8^ cells ml^−1^ across all categories of severity. Frogs with moderate infection had significantly higher concentrations than frogs negative for *Bd* (ratio: 1.9; 95% CIs: 1.3, 2.6), with negative frogs having the lowest sperm concentration (EMM: 4.4×10^8^; 95% CIs: 3.4×10^8^, 5.6×10^8^). Frogs with moderate infection also had a significantly higher sperm concentration than frogs with high infection status, with an EMM of 6.1×10^8^ cells ml^−1^ (95% CIs: 4.7×10^8^, 7.8×10^8^) and ratio of 1.4 (95% CIs: 1.0, 1.9). Frogs with high infection had significantly higher sperm concentration than frogs with no infection (ratio: 1.4, 95% CIs: 1.0, 2.0).

There was an effect of *Bd* severity on sperm forward-progressive motility (LRT χ^2^_2_=5.2, *P*=0.07) and total motility (LRT χ^2^_2_=5.1, *P*=0.08), with decreasing motility as infection severity increased (Fig. 1B,C). Odds ratios and 95% CIs were generated to further compare the differences in sperm quality. Frogs negative for *Bd* infection had the highest sperm motility, with 85.4% (95% CIs: 68.2, 94.1) forward-progressive motility and 91.7% (95% CIs: 84.0, 95.8) total motility. Frogs with moderate infection had 65.5% (95% CIs: 43.9, 82.2) sperm with forward-progressive motility and 82.3% (95% CIs: 71.0, 89.8) sperm with any motility. Frogs with high infection had the lowest motility, with 49.2% (95% CIs: 26.3, 72.5) sperm with forward-progressive motility and 75.3% (95% CIs: 59.8, 86.2) sperm with any motility. Sperm from uninfected frogs had significantly higher motility than frogs with high infection loads, with sperm from negative frogs having sixfold higher forward-progressive motility [odds ratio (OR): 6.0, 95% CIs: 1.5, 24.7] and almost fourfold total motility (OR: 3.6, 95% CIs: 1.3, 10.1). There was approximately a threefold (OR: 3.1, 95% CIs: 0.8, 11.7) and twofold difference (OR: 2.4, 95% CIs: 0.9, 6.3) in forward-progressive motility and total motility in negative frogs compared with frogs with moderate infection loads; however, this difference was not statistically significant. Frogs with moderate infection loads had a higher likelihood of forward-progressive motility (OR: 2.0, 95% CIs: 0.5, 7.5) and total motility (OR: 1.5, 95% CIs: 0.6, 4.0) than frogs with high infection loads; however, this difference was also not statistically significant.

There was no significant effect of *Bd* severity on sperm membrane integrity (LRT χ^2^_2_=0.4, *P*=0.8; Fig. 1D). Frogs negative for *Bd* infection had the highest membrane integrity EMM of 96.8% (95% CIs: 92.5, 98.7), followed by frogs with moderate infection load (EMM: 96.6%, 95% CIs: 92.6, 98.4) and frogs with high infection load (EMM: 95.3%, 95% CIs: 89.1, 98.0).

### Experiment 2: effect of initial *Bd* status on sperm quality pre- and post-treatment

**Fig. 2.**
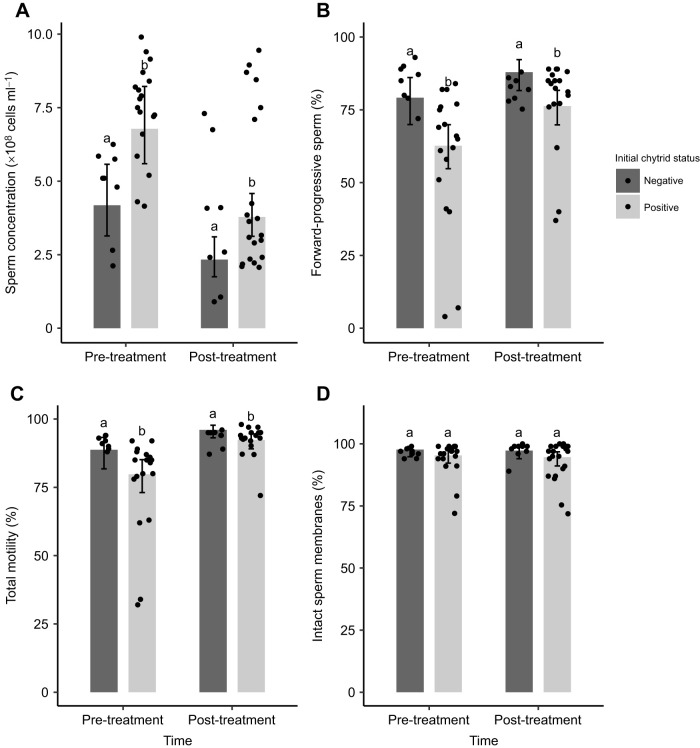
**Effect of initial *Bd* infection status and heat-treatment (pre- and post-) on induction of spermiation in *R. aurea* (*n=*13; negative, *n*=4 and positive, *n*=9).** (A) Sperm concentration (cells ml^−1^); (B) forward-progressive sperm (%); (C) total sperm motile (%); and (D) intact membranes (%). Columns represent estimated marginal means, black dots represent raw data and error bars equal 95% confidence intervals. Animals were sampled over both time points, and two technical replicates were made per frog, per timepoint. Repeated measures and technical replicates were included as random effects in the generalized linear mixed model. Different letters indicate significant differences among groups (*P*<0.05); groups sharing a letter are not significantly different.

There was a significant effect of initial infection status and heat treatment on sperm concentration (LRT χ^2^_1_=6.0, *P*=0.01 and LRT χ^2^_1_=622.8, *P*<0.001, respectively) after 5 weeks (Fig. 2A), with the highest sperm concentration in frogs initially testing positive for *Bd* pre-heat treatment. Both pre- and post-heat treatment, frogs that initially tested positive for *Bd* had significantly higher sperm concentration than frogs that initially tested negative, with the ratio of expected sperm concentrations being 1.6 (95% CIs: 1.2, 2.3). Irrespective of initial *Bd* status, sperm concentration was significantly higher prior to heat treatment than post-heat treatment, with the ratio of expected sperm concentrations being 1.8 (95% CIs: 1.7, 1.9), though within negative and positive groups there were no significant difference pre- or post-heat treatment (Fig. 2). Sperm concentration for positive frogs was 6.8×10^8^ cells ml^−1^ (95% CIs: 5.6×10^8^, 8.2×10^8^) and 3.8×10^8^ cells ml^−1^ (95% CIs: 3.1×10^8^, 4.6×10^8^) prior to and post-heat treatment, respectively. Sperm concentration for negative frogs was 4.2×10^8^ cells ml^−1^ (95% CIs: 3.1×10^8^, 5.6×10^8^) and 2.3×10^8^ cells ml^−1^ (95% CIs: 1.8×10^8^, 3.1×10^8^) prior to and post-heat treatment, respectively.

There was a significant effect of initial infection status and heat treatment on forward-progressive motility (LRT χ^2^_1_=6.0, *P*=0.01 and LRT χ^2^_1_=98.9, *P*<0.001, respectively). There was a weak effect of initial infection status, and a significant effect of heat treatment on total motility (LRT χ^2^_1_=3.6, *P*=0.06 and LRT χ^2^_1_=159.2, *P*<0.001, respectively). The highest forward-progressive motility and total motility was in frogs initially testing negative for *Bd*, post-heat treatment. In contrast to sperm concentration results, irrespective of whether disease had been treated, frogs that initially tested negative to *Bd* had significantly higher forward progressive (OR: 2.3, 95% CIs: 1.3, 4.1) and total motility (OR: 2.0, 95% CIs: 1.0, 3.9) than frogs which tested positive. Irrespective of initial disease status, frogs which were post-heat treatment for *Bd* had significantly higher forward-progressive (OR: 1.9, 95% CIs: 1.7, 2.2) and total motility (OR: 3.0, 95% CIs: 2.5, 3.7) than pre-treatment frogs. However, within negative and positive groups there were no significant difference pre- or post-heat treatment (Fig. 2). Frogs initially testing negative for *Bd* had higher forward-progressive sperm motility both pre- (79.2%, 95% CIs: 70.0, 86.1) and post-treatment (87.9%, 95% CIs: 81.6, 92.3), compared with 62.7% (95% CIs: 54.8, 69.9) and 76.3% (95% CIs: 69.8, 81.7), respectively, of frogs initially testing positive (Fig. 2B). Frogs post-heat treatment for *Bd* had higher total sperm motility for both frogs initially negative (96.0%, 95% CIs: 93.1, 97.73) and positive (92.3%, 95% CIs: 89.1, 94.7), compared with 88.7% (95% CIs: 81.8, 93.3) and 79.8% (95% CIs: 73.1, 85.1), respectively, in frogs pre-treatment (Fig. 2C).

There was no effect of initial infection status or heat treatment on membrane integrity (LRT χ^2^_1_=1.92, *P*=0.16 and LRT χ^2^_1_=1.72, *P*=0.18, respectively). Sperm membrane integrity ranged from 94.6% to 97.7% across all treatment groups (Fig. 2D).

### Experiment 3: chronic effect of heat treatment on longer-term sperm quality

**Fig. 3.**
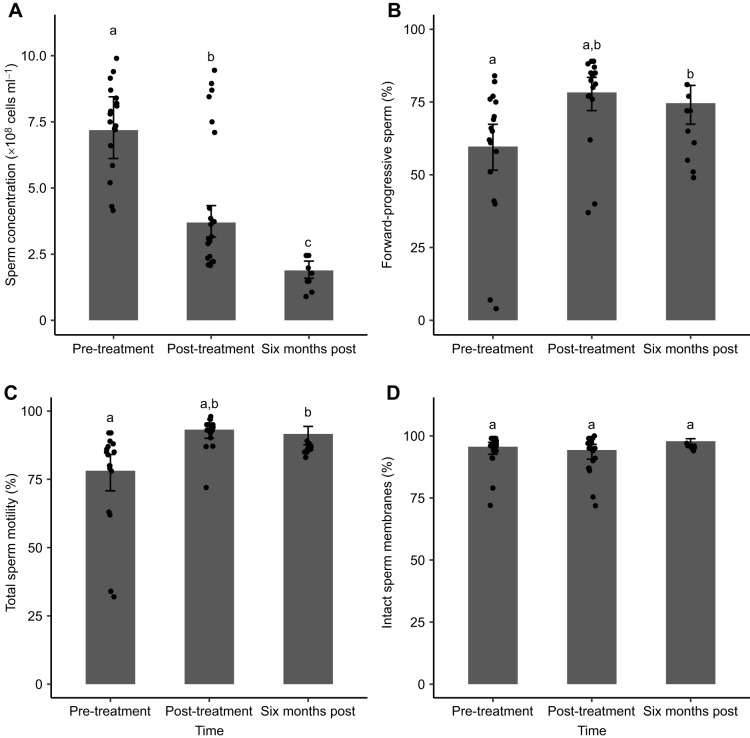
**Effect of time since heat treatment on induction of spermiation in *R. aurea* (*n*=4; pre-heat treatment, 5** **weeks post-heat treatment and 6** **months post-heat treatment).** (A) Sperm concentration (cells ml^−1^); (B) forward-progressive sperm (%); (C) total sperm motile (%); and (D) intact membranes (%). Columns represent estimated marginal means, black dots represent raw data and error bars equal 95% confidence intervals. Animals were sampled over both time points, and two technical replicates were made per frog, per time point. Repeated measures and technical replicates were included as random effects in the generalized linear mixed model. Different letters indicate significant differences among groups (*P*<0.05); groups sharing a letter are not significantly different.

In the four initially *Bd*-positive frogs monitored 6 months post-infection, there was a significant effect of time since exposure to heat treatment on sperm concentration (LRT χ^2^_2_=1150.1, *P*<0.001), with decreasing sperm concentration at both the 5-week and 6-month post-heat treatment time points (Fig. 3A). EMMs ranged from 1.9×10^8^ to 7.2×10^8^ cells ml^−1^ across all treatments. Frogs had the highest sperm concentration prior to heat treatment (EMM: 7.2×10^8^; 95% CIs: 6.1×10^8^, 8.5×10^8^). Expected sperm concentration prior to heat treatment was 2 times higher than 5 weeks post-heat treatment (ratio: 1.95; 95% CIs: 1.8, 2.1) and 4 times higher than 6 months post-heat treatment (ratio: 3.8; 95% CIs: 3.5, 4.1). Frogs 6 months post-heat treatment had lower sperm concentration (EMM: 1.9×10^8^, 95% CIs: 1.6×10^8^, 2.2×10^8^) than 5 weeks post-heat treatment (EMM: 3.7×10^8^; 95% CIs: 3.2×10^8^, 4.3×10^8^), with sperm concentration 2 times higher at 5 weeks post-heat treatment compared with 6 months post-heat treatment (ratio: 1.9; 95% CIs: 1.8, 2.1).

There was a significant effect of time since exposure to heat treatment on forward-progressive motility (LRT χ^2^_2_=151.2, *P*<0.001), and on total motility (LRT χ^2^_2_=206.0, *P*<0.001). For both forward-progressive motility and total motility, there was an increase in sperm quality 5 weeks post-heat treatment. Frogs had the lowest forward-progressive (EMM: 59.7%; 95% CIs: 51.6, 67.3) and total motility (EMM: 78.2; 95% CIs: 70.8, 84.1) counts prior to heat treatment. Frogs had the highest forward-progressive (EMM: 78.3%; 95% CIs: 72.0, 83.5) and total motility (EMM: 93.2%; 95% CIs: 90.0, 95.43) counts 5-weeks post-heat treatment, with a two-fold increase in forward-progressive motility (OR: 2.4; 95% CIs: 2.1, 2.8) and four-fold increase in total motility (OR: 3.8; 95% CIs: 3.1, 4.7) compared with pre-heat treatment (Fig. 3B,C). There was a decrease in both forward-progressive and total motility 6 months post-heat treatment compared with 5 weeks post-heat treatment, however, higher than prior to heat treatment. Forward-progressive motility was 74.6% (95% CIs: 67.4, 80.7) 6 months post-heat treatment. There was a two-fold increase in forward-progressive motility 6 months post-heat treatment compared with pre-heat treatment (OR: 2.0; 95% CIs: 1.6, 2.4). Forward-progressive motility 6 months post-heat treatment was higher than that 5 weeks post-heat treatment, though this difference was only just significant and minimal (OR: 1.2; 95% CIs: 1.0, 1.5). Total motility was 91.6% (95% CIs: 87.7, 94.4) 6 months post-heat treatment. There was a three-fold increase in total motility 6 months post-heat treatment compared with pre-heat treatment (OR: 3.1; 95% CIs: 2.4, 3.9). Total motility 5 weeks post-heat treatment was higher than that 6 months post-heat treatment, though this difference was only just significant and minimal (OR: 1.3, 95% CIs: 1.0, 1.6). There was a significant effect of time since exposure to heat treatment on sperm membrane integrity (LRT χ^2^_2_=29.4, *P*<0.001). However, membrane integrity ranged from 94.3% to 97.9% across all treatments, which is not considered biologically relevant (Fig. 3D).

### Sperm quality from deceased frogs

To determine whether *Bd* infection induced mortality causes complete loss of gamete quality, sperm was opportunistically collected post mortem from two frogs that died from *Bd* infection. These frogs had variable sperm quality metrics ranging in concentration from 1.39 to 2.43×10^8^ cells ml^−1^, 18.5–71.5% forward-progressive motility, 35.5–77.5% total motility and 79–79.5% membrane integrity (Table 1).

**Table 1.** Sperm quality from deceased male *Ranoidea aurea* (*n*=2)

	Sperm concentration (cells ml^−1^)*	Forward-progressive motility (%)	Total motility (%)	Membrane integrity (%)
Frog 1	2.43×10^8^±2.12×10^7^	71.5±2.1	77.5±0.7	79±0
Frog 2	1.39×10^8^±4.95×10^6^	18.5±3.5	35.5±0.7	79.5±0.7

*Equivalent to total number of sperm recovered from testes (since recovered into 1 ml SAR).

Data are means±s.d. of two technical replicates per macerate.

## DISCUSSION

This study examined how infection with *Bd* and subsequent thermal treatment influenced sperm quality and reproductive investment in male *R. aurea*. Frogs exposed to *Bd* exhibited variable infection loads and reproductive responses, with moderate infection associated with increased sperm concentration, while severe infection led to pronounced declines in both sperm concentration and motility. Thermal treatment successfully cleared the infection but reduced sperm quality across all individuals, indicating that the process of disease clearance imposed additional physiological costs. Six months after treatment, sperm motility and membrane integrity showed partial recovery, but sperm concentration remained significantly reduced, suggesting lasting impairment to spermatogenic function rather than transient suppression. These findings reveal that infection and thermal stress may interact to shape reproductive outcomes, underscoring the need to consider both disease and temperature dynamics in amphibian management and conservation.

The observed patterns in this study align with predictions from life-history theory, particularly the terminal investment hypothesis, which proposes that organisms facing elevated mortality risk will increase immediate reproductive effort to maximize fitness before death (Clutton-Brock, 1984; Williams, 1966). This study adds to the limited literature testing this hypothesis in amphibians infected with *Bd*. The increase in sperm concentration among moderately infected frogs, though only moderately higher, may suggest an upregulation of reproductive effort consistent with terminal investment as an acute response to infection. However, as infection severity intensified, sperm concentration and motility declined, indicating likely energetic trade-offs between immune defence and reproductive investment. The persistent reduction in sperm concentration post-treatment further supports the hypothesis that the energetic costs of immune activation, thermal stress and impacts of disease itself (e.g. failing osmoregulation) may produce enduring reproductive deficits that overwhelm the capacity of an organism to generate a terminal investment (Voyles et al., 2009). Similar context-dependent responses have been reported in other amphibians, where *Bd* infection increased reproductive output in some species but reduced it in others (Brannelly et al., 2016, 2021). These differences likely reflect variation in infection severity, life stage and exposure history, consistent with the dynamic threshold model proposed by Duffield et al. (2017), which predicts that terminal investment occurs along a continuum shaped by survival threat and residual reproductive value. Reductions in forward-progressive and total motility were present, but less defined. Although the reductions in this study may or may not be biologically relevant, they are still incredibly important considerations when considering that effects may be more pronounced with repeat exposures to infection.

In this study, even frogs that succumbed to *Bd* had viable and motile sperm within the testes post mortem, indicating that terminal effects of the disease are not necessarily equivalent to terminal disruption of gamete function, even if the possibility of deploying those gametes is prevented by death. Although this suggests that *Bd* infection does not pose a barrier to successful reproduction in this species, there remains a paucity of data regarding the interaction of various factors, such as population-wide exposure to disease, individual infection history, age and genetic diversity. Additionally, these data give support to post mortem sampling in this species for the purpose of biobanking, which can preserve genetic diversity, allowing lost alleles to be restored within populations (Calatayud et al., 2024).

The reproductive responses observed in *R. aurea* in this study differ in both magnitude and pattern from those reported in some other *Bd*-affected amphibians. Some species have shown increased sperm production and testicular development under infection, interpreted as evidence of terminal investment (Brannelly et al., 2016, 2021, 2025). However, apparent increases in reproductive activity under mild infection may also reflect inflammatory or stress-mediated effects rather than sustained adaptive investment. Infection-induced inflammation within the testes can alter cytokine profiles, disrupt Sertoli cell function and cause premature germ-cell release, leading to transient increases in sperm concentration or testicular activity that are not indicative of enhanced reproductive capacity (Dutta et al., 2021; Guazzone et al., 2009; Hedger, 2011). Although inflammatory responses in the skin may not directly lead to inflammation responses within the testis, there may be other indirect effects (e.g. loss of appetite or endocrinological changes) that lead to increased inflammation within the testis and/or to reduced reproductive output. The findings in *R. aurea*, where moderate infection was associated with increased sperm concentration, but severe infection and post-treatment conditions led to marked declines, support this interpretation. This suggests that what appears as terminal investment under mild infection may instead arise from short-lived inflammatory or stress-mediated effects that precede spermatogenic exhaustion, warranting further investigation. Such an acute pro-inflammatory response would only be adaptive if it could be demonstrated that fertilization rates or offspring survival and fitness were increased.

The reduction in sperm quality observed following thermal treatment aligns with known temperature-dependent disruptions of spermatogenesis. Even modest increases in testicular temperature can impair germ-cell differentiation and reduce sperm motility and concentration through oxidative stress, mitochondrial dysfunction, and apoptosis (López-Galindo et al., 2019; Maroto et al., 2025). Thermal exposure destabilizes the blood–testis barrier and triggers the production of reactive oxygen species, leading to germ-cell loss and DNA fragmentation. The partial recovery of motility but not concentration suggests that some aspects of sperm function may be reversible once thermally induced oxidative stress subsides, whereas damage to germ-cell populations or supporting Sertoli cells likely imposes longer-term limits on sperm production. Interestingly, membrane integrity was not significantly altered immediately following treatment, indicating that thermal and inflammatory stress primarily affected spermatogenic and maturation processes rather than post-spermatogenic membrane stability. These findings are consistent with oxidative and cytokine-mediated disruption of germ cell differentiation, rather than generalized cellular damage.

Over time, there was decreased sperm quality in frogs initially testing positive for *Bd*. The individuals initially testing negative could not be sampled at the 6 month time point. Thus, future studies may investigate whether long-term reproductive impacts were driven primarily by heat exposure or residual effects from infection itself. Regardless, these findings highlight that even after pathogen clearance there may be ongoing physiological consequences that reduce fertility and reproductive success in the short to medium term. Although thermal stress in this study was experimentally imposed to clear *Bd* infection and evaluate the reproductive costs of disease treatment, it also provides an important entry point for understanding how elevated temperatures associated with climate change may influence amphibian reproductive capacity. Similar physiological pathways are likely activated under natural heat extremes, suggesting that oxidative stress, mitochondrial dysfunction and impaired spermatogenesis may also operate in wild populations experiencing more frequent and intense thermal events. These pathways should be investigated further, as this overlap reinforces the value of using controlled thermal treatments not only as a disease-management tool but also as a model for assessing the broader impacts of climate warming on amphibian fertility and resilience. Although these results are only preliminary, they warrant further investigation. When viewed in the broader context of environmental change, these results indicate that the interplay between thermal stress, disease and reproductive physiology may represent a major constraint on amphibian resilience.

Evidence from hormonally induced spermiation studies indicates that sperm output can fluctuate in a sinusoidal or multi-peaked pattern across hours following induction, with concentration and motility rising and falling in recurrent waves rather than following a single peak or steady decline (e.g. Calatayud et al., 2025a). Broader reproductive studies have demonstrated that gametogenic and endocrine processes in amphibians also follow cyclical rhythms across seasonal and environmental gradients (Calatayud et al., 2018, 2022, 2025b preprint). The recognition of these temporal dynamics reinforces the need for future studies to incorporate repeated sampling across hours, days and reproductive seasons to more accurately characterize the duration, magnitude and variability of gametic responses to environmental and disease stress. Integrating this temporal resolution with established measures of reproductive quality will be essential for distinguishing transient stress responses from sustained shifts in reproductive allocation and for refining our understanding of amphibian resilience under changing climatic and pathogenic conditions.

### Conclusions

This study highlights how infection, temperature and reproduction intersect to shape the physiological resilience of amphibians facing environmental change. The findings indicate that what may appear as adaptive reproductive upregulation under mild infection can instead represent short-lived physiological adjustments, and that the long-term effects of disease and recovery depend on the timing and duration of reproductive cycles. The persistence of reproductive impairment after pathogen clearance underscores that disease mitigation may itself impose physiological costs, emphasizing the importance of understanding how reproductive processes fluctuate over time. Future studies should compare the reproductive response of males, females and offspring of naturally recovering and immune frogs (where mortality is not the endpoint) with susceptible species. Additionally, future comparisons should also monitor the effects of emerging treatments/vaccination regimes, including antifungals and controlled exposure to the pathogen itself, on gamete quality and fertilisation success. More broadly, these results point to the need for integrated research that links pathogen management, thermal tolerance and reproductive biology, aligning with the priorities set out in the Amphibian Conservation Action Plan (Calatayud et al., 2024). By bridging these disciplines, conservation physiology can better predict how disease and climate stress jointly constrain fertility, adaptation and long-term population persistence in a rapidly changing world.
